# Prediction of DNA-binding protein based on statistical and geometric features and support vector machines

**DOI:** 10.1186/1477-5956-9-S1-S1

**Published:** 2011-10-14

**Authors:** Weiqiang Zhou, Hong Yan

**Affiliations:** 1 Department of Electronic Engineering, City University of Hong Kong, Kowloon, Hong Kong; 2 School of Electrical and Information Engineering, University of Sydney, NSW2006, Australia

## Abstract

**Background:**

Previous studies on protein-DNA interaction mostly focused on the bound structure of DNA-binding proteins but few paid enough attention to the unbound structures. As more new proteins are discovered, it is useful and imperative to develop algorithms for the functional prediction of unbound proteins. In our work, we apply an alpha shape model to represent the surface structure of the protein-DNA complex and extract useful statistical and geometric features, and use structural alignment and support vector machines for the prediction of unbound DNA-binding proteins.

**Results:**

The performance of our method is evaluated by discriminating a set of 104 DNA-binding proteins from 401 non-DNA-binding proteins. In the same test, the proposed method outperforms the other method using conditional probability. The results achieved by our proposed method for; precision, 83.33%; accuracy, 86.53%; and MCC, 0.5368 demonstrate its good performance.

**Conclusions:**

In this study we develop an effective method for the prediction of protein-DNA interactions based on statistical and geometric features and support vector machines. Our results show that interface surface features play an important role in protein-DNA interaction. Our technique is able to predict unbound DNA-binding protein and discriminatory DNA-binding proteins from proteins that bind with other molecules.

## Background

More and more structural data are becoming available for biomolecules, which provide valuable resources for the study of biomolecular interactions. In recent years scientists have made a lot of efforts in studying protein-DNA interactions based on X-ray crystallography and NMR data. Samudrala and Moult proposed an all-atom distance-dependent discriminatory function for the prediction of nucleic acid binding proteins [1]. Later, Moont et al. applied an interface pairwise residue level potential to the screening of predicted docked complex [2]. Recently, Robertson and Varani improved the method based an interface-atom distance-dependent formalism and showed that this obtained better prediction power than previous methods [3]. However, these studies mainly focussed on the bound structure of DNA-binding protein. The ability to recognize DNA-binding sites in structures that are unbound is potentially useful. There are several other methods that have been devised to predict protein-related interactions: Lo et al. used residue contacts to study the helix-helix interaction in membrane proteins [4], Xu et al. applied collective matrix factorization in the prediction of protein-protein interaction [5], Gonzalez et al. introduced correlation analysis based method in the prediction of protein-ligand interaction [6], Ahmad proposed the usage of moment information in the prediction of DNA-binding proteins [7].

Previous attempts to predict protein-DNA interaction provided acceptable results, but the underlying principle of protein-DNA interactions is not fully understood. As the progress made in genomic projects, more high resolution 3D structures of biological molecules have become available now. However, few of the previous studies paid enough attention to the 3D interface surface characteristics of the protein-DNA complex. Interface surface characteristics such as atom type, residue type, surface curvature, accessible surface area, etc. play important roles in protein-DNA interaction. In order to analysis the properties of the molecules, a 3D model is need. Alpha shape has been proved to be useful in molecule analysis and has been used for in molecular volume computation, cavities detection and shape representation. Liang et al. first proposed to use alpha shape modeling to compute the molecular area, volume and to detect the inaccessible cavities in proteins [8,9]. Li et al. used the edges in alpha shape modeling to represent the protein structure and atom contacts [10]. Poupon used Voronoi tessellations to compute the protein volume and detect the pockets, cavities and voids on the protein surface [11]. Recently, alpha shape has been introduced into the study of molecular surfaces. Albou et al. applied alpha shape modeling to characterize the surface of the proteins and defined the surface residue and surface patches [12]. Zhou and Yan applied alpha shape modeling and conditional probability in the study of protein-DNA interface properties [13,14].

In this, we propose to apply 3D alpha shape modeling to study the interface surface characteristics of the protein-DNA complex and develop an algorithm for the prediction of unbound DNA-binding proteins based on statistical and geometric features and support vector machine.

## Methods

### Data selection

Trainset: This training dataset contains 199 DNA-binding proteins with different functions constructed by Zhou and Yan [13] to serve as the correct structure data. The control data contains 86 RNA-binding proteins, 106 ligand-binding proteins [13] and 186 protein-binding proteins [15].

Testset: The testing dataset contains 104 unbound DNA-binding proteins and 401 non-DNA-binding proteins as per Zhou and Yan [13].

TemLib: The correct protein-DNA complex dataset contains the same 199 different types of DNA-binding proteins used in Trainset which form the template library for structural alignment.

### Alpha shape modeling

Alpha shape modeling has been used to study the molecular structures such as the detection of pockets in known structures, computation of the molecular volume and description of the protein surface [11,12]. In this study, we apply the 3D alpha shape model to the reconstruction of the interface of protein-DNA interaction and used geometric features to represent the properties of protein-DNA interaction.

**Figure 1:**
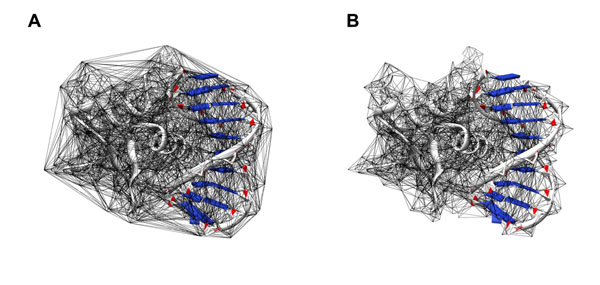
**Alpha shape construction** (A) The edges of the Delaunay triangulation of a protein-DNA complex. (B) The edges of the alpha shape obtained from the Delaunay triangulation.

We can construct the 3D alpha shape based on the Delaunay triangulation [16]. The alpha shape is a subset of the tetrahedrons in the Delaunay triangulation complex which is a generalization of the convex hall of the point set [17] (the atoms in a molecule). Comparison between the edges of the Delaunay triangulation of a protein-DNA complex and the edges of the alpha shape is shown in Figure 1. We can see from Figure 1(B) that the alpha shape can be created by trimming the edges of the Delaunay triangulation.

Theoretically, the alpha shape can be computed as follows. First, we define the alpha complex based on a set of points {*P*}. Given the value of *α*, the alpha complex includes all the simplexes in the Delaunay triangulation which have an empty circumsphere (‘empty’ means that the open sphere does not include any points of {*P*}) with a squared radius equal to, or smaller than, *α*. The alpha shape can be obtained by the domain covered by the simplexes of the alpha complex. Notice that, the alpha value here actually controls the roughness of the molecular surface obtained. In this work, we use the CGAL [18] library to compute the alpha shape.

An important step before extracting geometric features from the alpha shape model is the definition of interface surface which contains most of the information need to predict protein-DNA interaction. In the alpha shape model, the vertices correspond to the surface atoms of the original protein-DNA structure. Using this characteristic, the interface atoms of the protein-DNA structure can be defined as follows. First, we calculate the alpha shape of the protein-DNA complex and obtain the surface atom set {*Complex*}. Then, we calculate the alpha shape of the protein independently (with the DNA part removed) and obtain the protein surface atom set {*Protein*}. Finally, the interface atoms set can be obtained by retaining the atoms which are in {*Protein*} but not in {*Complex*}.

### Structural alignment

**Figure 2:**
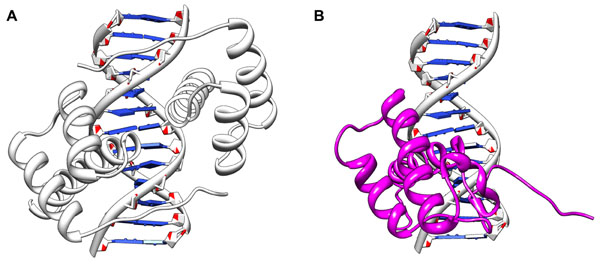
**Structural alignment process** (A) The template DNA sequence with its interacting protein structure. (B) The new structure formed by the target protein structure and the template DNA sequence.

In order to predict the potential interface between the unbound proteins and DNA, we make use of structural alignment. For a target protein structure, for which we would like to predict whether it will bind to any DNA, we carry out structure alignment against the template library (TemLib) using TM-align [19]. The target structure is scanned against every structure in the template library to find the most similar structure as defined by the largest TM-scored [19]. In Testset, any template with a sequence overlap larger than 35% with another template is excluded from the template library to avoid the over-training problem. After structural alignment, a new protein-DNA structure is created by replacing the selected template sequence with the aligned target protein structure (as shown in Figure 2).

### Statistical and features

#### Residue Index

Considering the specificity of the interface residue types, we define a feature-named residue index by computing the percentage of each residue type showing up in the interface for each interface atom set defined above. All 20 residue types are considered and a residue index is represented as a vector in the following format: (ALA%, ARG%, ASN%, ASP%, CYS%, GLU%, GLN, GLY%, HIS%, ILE%, LEU%, LYS%, MET%, PHE%, PRO%, SER%, THR%, TRP%, TYR%, VAL%).

#### TM-score

The TM-score is obtained using TM-align [19] in the structure alignment process. It is a measure of the similarity between the template protein structure and the target protein structure.

#### Curvature Index

In order to characterize the interface properties geometrically, we calculate the interface surface curvature. It is derived from the solid angle Ω of the interface atoms in the alpha shape model, and it is transformed to the range of -1 (cleft) to 1 (knob) using cos(Ω/4). The curvature index is defined as a vector-containing convex- and concave index: (Convex Index, Concave Index). The convex index represents the overall knob level of the interface which is a summation of the positive solid angles in the interface, and it is normalized by the number of interface atoms. The concave index, on the other hand, represents the overall cleft level of the interface which is a summation of the negative solid angles, and it is also normalized by the number of interface atoms.

#### Mean Connectivity

The connectivity of the surface atoms in the molecule is introduced by Zhou and Yan [20]. The vertices of the alpha shape model correspond to the surface atoms of the molecule being reconstructed. The connectivity of a surface atom is defined as the connection between this atom and the other surface atoms, which correspond to the edges in the alpha shape model. Mean connectivity is computed by summing up the connectivity of all the interface atoms and normalized by the number of interface atoms.

#### Interface Atom Index

The interface atom index is defined by the percentage of the interface atom taken in the overall protein-protein complex, i.e., the ratio of the number of interface atoms to the number of atoms in the whole complex. It is a measure of the interface size relative to the complex size.

### Support vector machine (SVM)

SVM has been widely used in pattern classification problems. As a machine learning algorithm, SVM has to build the classification rules based on the existing knowledge. This type of learning method requires training data with known class labels. The input data of the SVM are vectors. In this sense, SVM is suitable for high dimensional data classification. In this study, we use the mySVM software [21] to predict protein-DNA interaction based on the statistical and geometric features.

### Performance evaluation

In order to evaluate the performance of the prediction result, we use different measures including Recall, Specificity, Precision, Accuracy and Matthews correlation coefficient (MCC). First, we define TP (true positive, the number of proteins correctly predicted as DNA-binding), FP (false positive, the number of proteins incorrectly predicted as DNA-binding), TN (true negative, the number of proteins correctly predicted as non-DNA-binding) and FN (false negative, the number of proteins incorrectly predicted as non-DNA-binding). The definitions of the measures are as follows:

Recall: *TP*/(*TP* + *FN*);

Specificity: *TN*/(*TN* + *FP*);

Precision: *TP*/(*TP* + *FP*);

Accuracy: (*TP* + *TN*)/(*TP* + *FN* + *TN* + *FP*);

MCC: .

## Results

In order to evaluate the performance of the proposed method, we perform two experiments to discriminate the unbound DNA-binding proteins from the unbound non-DNA-binding proteins in Testset using the proposed method and the discriminatory function developed by Zhou and Yan [13] respectively.

### Performance of SVM classifier

Structural alignment is carried out using TemLib as the template library for the Trainset and Testset. Then we construct the alpha shape model of the new structures based on CGAL [18]. The alpha value is set to be the value to obtain one connected component automatically in CGAL. Features are extracted from the potential DNA-binding interface surface information of the proteins. A vector of 25 dimensions is used to represent each structure in the following format: {residue index, TM-score, curvature index, mean connectivity, interface atom index}.

The pattern recognition SVM is used with the radial kernel implemented in mySVM. When the parameters *C*, *γ* and elision+ are set to 0.1, 0.01 and 0.5 respectively [21], mySVM produced a good classification performance for both sensitivity and specificity. The label for the correct data is set to 1 and the label for the control data is set to -1 for SVM training. Performance of the classification for Testset is shown in Table 1. Within 104 unbound DNA-binding proteins, 54 proteins are correctly recognized which results in a percentage accuracy for Recall of 43.27. All the other performance evaluation measures reach a very high value due to 392 in 401 unbound non-DNA-binding proteins being correctly recognized. The overall performance is; Precision, 83.33%; Accuracy, 86.53%; and MCC 0.5368. These results demonstrate that the proposed method shows good performance in the classification of non-DNA-binding proteins from the DNA-binding proteins.

**Table 1:** Comparison of performance between the conditional probability discriminatory function (CPDF) and SVM classifier (SC).

Method	Recall	Specificity	Precision	Accuracy	MCC
CPDF (-1.7)	48.08%	70.57%	29.76%	65.94%	0.1601
CPDF (-2.3)	44.23%	82.29%	39.32%	74.46%	0.2542
SC	43.27%	97.76%	83.33%	86.53%	0.5368

### Performance comparison with the conditional probability discriminatory function

In the conditional probability discriminatory function developed by Zhou and Yan [13], three features are considered: atom type, residue type and surface curvature. They used the conditional probability method to construct a scoring function for the potential DNA-binding proteins. To be consistent with the results in [13], we use the same training data and choose 200 for the number of bins and 14 for the alpha value as mentioned in their work. (The related data set and program are obtained from http://www.hy8.com/bioinformatics.htm).

According to their method, the best classification can be obtained by setting the threshold rage from -1.7 to -2.3. We use -1.7 and -2.3 to compute the performance evaluation measure mentioned in the Methods section. The result is shown in Table 1.

## Discussion

Comparing the results of the two methods, we can see that both methods show good performance in recognizing DNA-binding proteins. The conditional probability discriminatory function (CPDF) shows better performance in recognizing the DNA-binding proteins than the SVM classifier (SC) in terms of Recall. However, in terms of Specificity and Precision, SC outperforms CPDF in the discrimination of non-DNA-binding proteins. The reason is that the method developed in this study considers a wider range of interface surface features. This result shows that interface surface features are dramatically different between DNA-binding proteins and non-DNA-binding proteins. Accuracy and MCC measures the overall performance of the classifier, we can see that due to the low false positive rate these two measures of SC are much better than that of CPDF. From this comparison, we can see that the method developed in this study shows comparable ability in recognizing the DNA-binding proteins with the previous method [13] and outperforms the previous method in recognizing non-DNA-binding proteins.

## Conclusions

In this paper, we propose a method based on structural alignment to predict protein-DNA interaction. TM-align is used to search for proper template structures in structural alignment. New structures are made by replacing the original protein structures with the target proteins. We apply a 3D alpha shape model to represent the interface of the protein-DNA complex, and we extract statistical and geometric features including residue index, TM-score, curvature index, mean connectivity and an interface atom index to characterize the interface properties. The SVM is used to classify DNA-binding proteins and non-DNA-binding proteins using the interface features. The performance of our method is tested by discriminating 104 DNA-binding protein structures from 401 non-DNA-binding proteins. We use different evaluation measures to represent the experiment output of our method. In the experiment, we have achieved an accuracy of 86.53% and an MCC of 0.5368. Our method outperforms the conditional probability discriminatory function in terms of MCC, which indicates the good performance achieved by our method in discriminating between DNA-binding proteins and non-DNA-binding proteins. This study shows that interface surface features play an important role in protein-DNA interaction. Our method is able to predict unbound DNA-binding proteins and discriminatory DNA-binding proteins from proteins that bind with other molecules. The result indicates the potential usage of the proposed method in the study of protein and other related fields.

## Competing interests

The author(s) declare that they have no competing interests.

## Authors' contributions

WQ carried out the programming and analysis work. HY initiated the project and provided suggestions on the formulations based on alpha shapes. Both authors read and approved the final manuscript.
